# Submucous Fibroids, Fertility, and Possible Correlation to Pseudocapsule Thickness in Reproductive Surgery

**DOI:** 10.1155/2018/2804830

**Published:** 2018-09-03

**Authors:** Andrea Tinelli, Ioannis Kosmas, Ospan A. Mynbaev, Alessandro Favilli, Grigoris Gimbrizis, Radmila Sparic, Marcello Pellegrino, Antonio Malvasi

**Affiliations:** ^1^Department of Obstetrics and Gynecology, Division of Experimental Endoscopic Surgery, Imaging, Technology and Minimally Invasive Therapy, Vito Fazzi Hospital, P.zza Muratore, Lecce, Italy; ^2^Laboratory of Human Physiology, Phystech BioMed School, Faculty of Biological & Medical Physics, Moscow Institute of Physics and Technology (State University), Dolgoprudny, Moscow Region, Russia; ^3^Department of Obstetrics and Gynecology, University of Ioannina, Greece; ^4^Division of Molecular Technologies, Research Institute of Translational Medicine, N.I. Pirogov Russian National Research Medical University, Moscow, Russia; ^5^Institute of Numerical Mathematics, RAS, Moscow, Russia; ^6^Department of Obstetrics and Gynaecology, University of Perugia, Perugia, Italy; ^7^1st Department of Obstetrics and Gynecology, Aristotle University of Thessaloniki, Greece; ^8^Clinical Centre of Serbia, Clinic for Gynecology and Obstetrics and University of Belgrade, School of Medicine, Belgrade, Serbia; ^9^Division of Human Pathology, Vito Fazzi Hospital, Lecce, Italy; ^10^Department of Obstetric & Gynecology, Santa Maria Hospital, GVM Care & Research, Bari, Italy

## Abstract

**Background and Objectives:**

Fibroids are related to infertility. Fibroid pseudocapsule is a neurovascular bundle surrounding leiomyomas rich of neurofibers involved in myometrial biology. Authors evaluated, by a case-control study, the fibroid pseudocapsule (FP) thickness by ultrasound (US) and the histological measurements, according to uterine location of fibroids.

**Methods:**

137 consecutive patients undergoing hysterectomy for uterine myomas were enrolled and 200 myomas were evaluated. Before surgery, patients underwent an ultrasound (US) investigation to evaluate the number, the size, and the location of fibroids. After surgery, myoma-pseudocapsule-myometrium specimens were measured and evaluated by a single expert pathologist. Both US and histological data were collected and statistically analyzed.

**Results:**

Our results confirm the relevant difference of FP thickness, particularly represented under the endometrium for submucous LMs. FPs near the endometrial cavity were considerably thicker than those of both intramural fibroids and subserous fibroids measured by US (P=0.0001) and histology (P=0.0001). A clear cut-off measurement at 2 mm (P=0.0001) was found between endometrial FPs and all other FPs for either US or histology measurements.

**Conclusion:**

The thickness of FP is considerably higher near the endometrial cavity when compared to those of both intramural and subserous LMs, suggesting a potential role either in fertility or in myometrial healing.

## 1. Introduction

Uterine fibroids or leiomyomas (LMs) are the most common worldwide indication for hysterectomy [1, 2]. Although mostly of women with fibroids are asymptomatic, LMs can cause abnormal uterine bleeding, pelvic pain, and reproductive dysfunction [1]. It is difficult to assess a correct uterine LMs incidence as it increases with ageing. They may occur in more than 30% of patients of 40-60 years [3]. Nowadays, uterine LMs represent not only a problem for the women health, but also a heavy economic burden. It has been estimated that the American social costs for uterine LMs, in terms of costs of care, adverse obstetric outcomes, and work-hours lost, are higher than that ovarian, breast, and colon cancer [4]. In the last decade, several pharmacological [5] and surgical treatments have been proposed for a conservative management of uterine LMs [6, 7].

In order to preserve fertility, a conservative treatment should be proposed to women wishing pregnancies, especially in those younger patients who want to undergo assisted reproductive techniques (ART). There is a general agreement that submucosal LMs negatively affect fertility, when compared to women without fibroids. A recent review reported that intramural LMs above a certain size (>4 cm), even without cavity distortion, may also negatively influence fertility and the presence of subserosal LMs has little or no effect on fertility [8].

Nevertheless, some studies reported conflicting results and much of the data shows no differences in outcomes no matter the size of fibroids. Vimercati et al. [9] affirmed that patients with fibroids >4 cm required an increased number of cycles to obtain an ongoing pregnancy, compared with the other groups. On the contrary, Oliveira et al. [10] concluded that patients with subserosal or intramural fibroids < 4 cm had IVF-ICSI outcomes (pregnancy, implantation, and abortion rates) similar to those of controls and women with intramural fibroids > 4.0 cm had lower pregnancy rates than patients with intramural fibroids ≤ 4.0 cm of diameter.

Yan et al. [11] showed that women with intramural fibroids with the largest diameter < 2.85 cm or the sum of reported diameters < 2.95 cm had a significantly higher delivery rate than patients with larger fibroids. A significant negative effect on delivery rate was noted when intramural fibroids with the largest diameter greater than 2.85 cm were considered, compared with matched controls without fibroids. Although noncavity-distorting fibroids do not affect IVF/ICSI outcomes, intramural fibroids greater than 2.85 cm in size significantly impair the delivery rate of patients undergoing IVF/ICSI. On the other side, Savarelos et al. [12] reported that women with intracavitary distortion and undergoing myomectomy significantly reduced their midtrimester miscarriage rates in subsequent pregnancies from 21.7 to 0% (P< 0.01). This result have been translated to an increase in the live birth rate from 23.3 to 52.0% (P< 0.05). Conversely, Yarali et al. [13] affirmed that the implantation and clinical pregnancy rates were similar on intramural and subserous fibroids (that did not distorted the uterine cavity). Horcajadas et al. [14] concluded their study with no correlation between implantation and miscarriage with leiomyoma number and size, although the focus of the study is in the gene expression and not on a comparative study between the position, size, and number of fibroids.

Trying to understand the correlation between LMs and fertility, some authors deeply studied the LMs anatomical and biological structure, in order to develop even more conservative and effective treatments [15, 16]. From the LMs anatomy studies, the neuroendocrine-biological role of the fibroid pseudocapsule (FP), a sort of neurovascular bundle surrounding LM, on myometrial physiology emerged [17]. Several studies have highlighted a new endocrine function of such structure, which may have a potential role in the uterine healing and fertility, especially after myomectomy [18–24]. Recently, a nontumoral origin of FP has been speculated, but rather a protective structure from the healthy myometrial tissue that could enhance regenerative mechanisms [25].

The FP is a well-known anatomical entity, which can be sonographically [17, 25] and histologically evaluated [26]. In a previous preliminary report [27], authors examined the pseudocapsule thickness according to uterine location of LMs, detecting a high correspondence between ultrasound (US) and the histological measurement. Nevertheless, the FP was considerably thicker over the submucous myomas when compared to those of both intramural and subserous LMs, suggesting a potential role in healing mechanism. The limits of such investigation involved a limited number of patients. Therefore, the aim of such prospective case-control study with single surgeon was to validate the results raised in the previous report and to assess the repeatability of the measurement techniques in a large cohort of patients.

## 2. Material and Methods

From 2009 to 2015, authors conducted a prospective single centre study conducted in Italian affiliated University Hospital, in a cohort of patients affected by fibroids and scheduled for hysterectomy. All selected patients consented to take part in research, as well as be operated. The study design was approved by the IRB. All procedures were in accordance with the guidelines of the Helsinki Declaration on human experimentation. All enrolled patients complained symptoms related to fibroids, such as heavy menstrual bleeding and pelvic pain. The surgical treatment was clinically indicated and the patient care was not altered by participation in this study.

A written, informed, and signed consent for hysterectomy was signed from all patients. Cases of endometrial hyperplasia, uterine polyps, cervical intraepithelial neoplasia, uterine or cervical cancer, confirmed or suspected primary adnexal pathology, adenomyoma, or adenomyosis were excluded from this study.

Fibroids were excluded from statistical analysis if they had been mapped as intraligamentary and/or in the isthmic-cervical region, as well as pedunculated.

Before surgery, patients underwent an ultrasound investigation in the first 10 days of the menstrual cycle to evaluate the number, the size, and the location of fibroids according to the LMs subclassification system of International Federation of Gynecology and Obstetrics (FIGO), with the following classification: Group 1: FIGO Classes 1&2, Group 2: FIGO Classes 3&4, and Group 3: FIGO Classes 5&6 [25].

Moreover, the pseudocapsule thickness (the white ring surrounding the myoma) was measured for each myomas following the methods described in the previous report [26].

The US examination and measurements were performed by a single US-expert (A.T.). The following US systems, a Logic 7 Pro US system (GE-Kretz, Zipf, Austria) or a Voluson 730 US system (GE-Kretz, Zipf, Austria) equipped with a 3.8 to 5.2 MHz transvaginal transducer, were used. Both machines were settled by the producer Industries with a medium-level quality, by a standard US setting of Doppler and gray scale.

The hysterectomies were performed both in laparoscopic or laparotomic setting at the first ten days of menstrual cycle. After surgery, myoma-pseudocapsule-myometrium specimens were measured and evaluated by a single expert pathologist (M.P.), blinded for patients' data. Pathologic analysis was carried out by the same methodology described in the previous report [26]. Afterwards both US and histological data were collected and send for statistical analysis to a member of this international research team, then all results were analyzed, and manuscript was drafted by three members of this team.

## 3. Statistical Analysis

FP measurements have been tested for normal distribution using Q-Q plots. Both LM thicknesses, measured by US and histology, were analyzed by the one-way ANOVA test. P value <0.05 was considered as statistically significant. By extending the ANOVA method, we used each pair of Student's T test (<0.05, all pairs Tukey-Kramer test (<0.05) comparison with Best Hsus MCB (<0.05) and Dunnett's (<0.05). Exploratory analysis was performed with partition with three splits, because no prior model existed. Pearson correlation was employed to find whether positive correlation exists between the two measurements, because data are normally distributed (Q-Q plots not seen). Area under the curve was performed with ROC curves. Analyses were conduct with the Statistical Package JMP 9 (SAS) and SPSS 15.0 (SPSS Inc., Chicago, IL, USA).

## 4. Results

One hundred and thirty-seven consecutive patients undergoing hysterectomy for LMs were enrolled in this study. Normal distribution was observed in the tree FIGO classification groups. The total enucleated LMs were 200: 62 fibroids in FIGO Classes 1&2, 73 in FIGO Classes 2&3, and 65 in FIGO Classes 5&6.

FPs near the endometrial cavity were considerably (P=0.0001) thicker than those of both intramural and subserous LMs measured by US (2.62 ± 0.31 versus 1.68 ± 0.13 and 0.97 ± 0.36 mm) and histology (2.75 ± 0.27 versus 1.72 ± 0.2 and 1.06 ± 0.4 mm), respectively.

**Figure 1:**
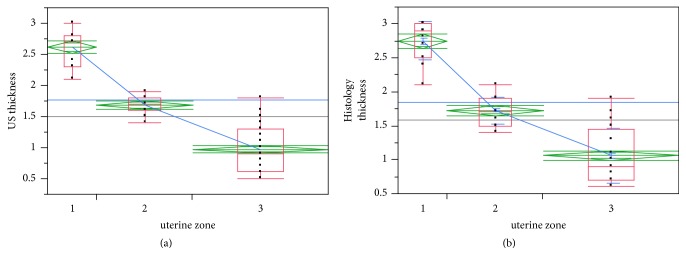
(a) The myometrial fovea after enucleation of the myoma; (b) the pseudocapsule with white fibro-connective bridges highlighted during hysteroscopic myomectomy.

Significant difference was observed between the three groups, for both measurements, using all tests mentioned above (Figures 1(a) and 1(b)).

**Figure 2:**
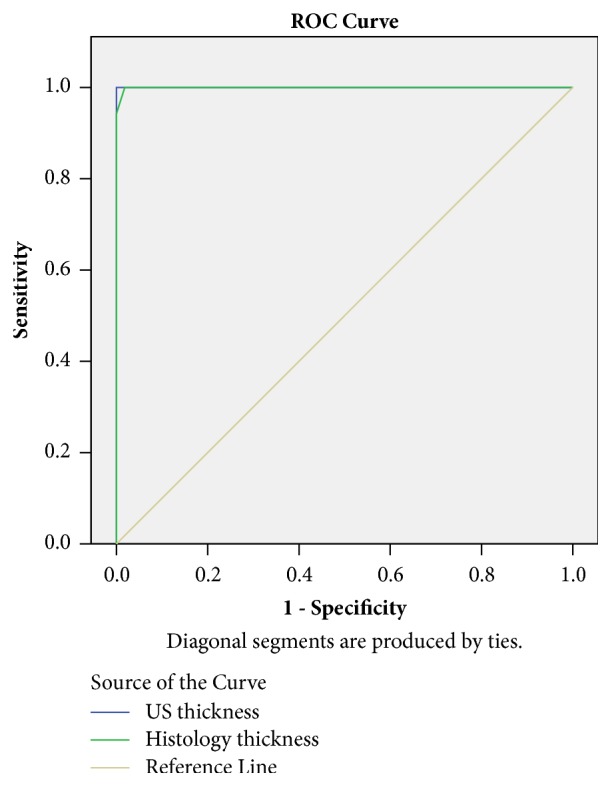
Area under the curve for US measurement (0.949) and histology measurements (0.953) for pseudocapsule thickness from fibroids of endometrial cavity location. Values are near 1, thus indicating that this test is of high accuracy for pseudocapsule measurements of endometrial cavity fibroids.

On exploratory analysis, a clear cut-off measurement at 2 mm (P=0.0001) was found between near the endometrium FPs and all other FPs for either US or histology measurements. Area under the curve was 0.949 for US and 0.953 for histology for endometrial cavity fibroids (Figure 2).

Correlation between ultrasound and histology measurements was near 1, indicating that ultrasound and histology measurements are positively correlated (0.954 P=0.000) (Pearson correlation).

## 5. Discussion 

Authors have found that the FP thickness was significantly different according to LMs uterine position. The FP of the submucous LMs appears considerably thicker in comparison than those of both intramural and subserous LMs. These features of FP depending their localization were observed both in presurgical US and in the histological examinations and US and histological measurements were highly correlated. A major strength of this study compared to the previous one [26] is the large cohort of involved patients.

Submucosal fibroids have a statistically significant negative effect on clinical pregnancy rates as reported by a meta-analysis of 13 studies [22]; the study also showed a lesser extent of intramural fibroids on clinical pregnancy rates. About delivery rates, submucosal and intramural fibroids showed a negative impact. On the contrary, subserosal myomas did not showed any effect on clinical pregnancy rates and delivery rates.

A meta-analysis of Pritts et al. [23] showed that fibroids are generally linked to a statistically significant decrease in fertility, regarding clinical pregnancy and birth rates and, simultaneously, an increase in miscarriage rates. The submucosal fibroids have the greatest negative statistical correlation on clinical pregnancy rates, so intramural fibroids resulted in significantly lower birth rates and higher miscarriage rates.

Pritts et al. [23] concluded that both patients with submucosal and intramural fibroids have poorer reproductive outcomes compared to patients without fibroids.

Thus, submucous and intramural LMs are more involved for sterility and infertility cases due to alteration of uterine cavity and contractility, while subserosal fibroids do not seem to generate any obvious fertility issue.

These surgical conclusions conflicted with studies focusing on endometrial receptivity in uteri with submucosal fibroids, showing surgical removal of intramural fibroids with no improvement in outcomes. Rackow et al. [28] reported that endometrial receptivity markers significantly decrease in submucosal fibroids, while the same is evident for intramural fibroids [29], especially for the HOXA10 gene. After intramural myomectomies a statistically significant increase was observed by Unlu et al. [30] in these receptivity markers, but unfortunately they did not observe such an effect in the submucosal myomectomies. Overall, only these two studies exist in the endometrial receptivity and myomectomy. Although evidence is still minimal, we assume that one factor of improved implantation rates after removal of intramural and submucosal fibroids is the improvement of implantation profile. Although, preservation of pseudocapsule achieves no early postoperative complications and good fertility rates [31], new studies need to be performed in the role of fibroid pseudocapsule preservation and the implantation markers.

From the other side, many other theories have been developed until now for the improvement of fertility rates after submucosal myomectomy. Horne et al. [32] reviewed the theory, as the mechanical distortion of the endometrial cavity, the disruption of the junctional zone within the myometrial layer, the altered vasculature due to the abnormal expression of angiogenic factors, the inflammation mediated changes in the endometrium, and, as lastly new, the alteration of endometrial receptivity factors.

In view of the above surgical evidences, we could correlate the greater thickness of the FP in the submucous and then in the intramural LMs. Among the possible theories which have been proposed in order to explain how fibroids may impair fertility [8], although we do not have a clear explanation of why there is an increase in thickness in LMs in submucous and intramural LMs, we must consider this evidence and further study it.

Our theories formulated on pseudocapsule thickness potential impact in fertility for future investigation consider mechanical reasons and differences in genetic expression components.

The pseudocapsule surrounding fibroids consist of compressed myometrium containing nerves and blood vessels that continue into adjacent myometrium [33]. Uterine stroma might not allow development of intramural pseudocapsule as in fibroids near endometrial cavity. In addition, one of the most frequently observed endometrial histological changes surrounding submucous LMs is glandular atrophy and ulceration, affecting also the proximal and the distal part of the endometrium over LMs [8]. It is possible that the thickest FP of the submucous LMs will be implicated in the endometrial modification that will consistently reduce the female fertility. FP growth of submucous LMs could reduce and adversely affect the overlying endometrium, becoming atrophic. What is not clear is whether increasing of the FP thickness should increase also the amount of normal quota of neuroendocrine fibers [17]. Normally, both protein gene product 9.5 (PGP9.5) and oxytocin demonstrated no significant differences in the density between the FP and adjacent normal myometrium, regardless of the fibroid location in the uterus. The neuroendocrine PGP9.5 immunoreactive nerve fibers may be involved in the pathophysiology of uterine LMs and affect muscle contractility, uterine peristalsis, and muscular healing.

From the other side, pseudocapsule vasculature present with disarray in vascular architecture with absence of vessel parallelism and variable intervascular distances. The different density of vessels per space indicated an abnormal vascular branching of pseudocapsule and some vascular walls without interruption indicated vessel tortuosity. There were vascular spaces, which did not communicate with other vessels (“cul-de-sac” vessels). All previous data present with geometrical characteristics of malignant neoplasm vessels [18]. From the other side, differences in the genetic profile are expressed between fibroids and adjacent endometrium. Angiogenesis promoters' expression is reduced when compared with myometrium while the precursor of angiogenesis inhibitor has reduced expression relative to endometrium. That explains the reduced microvascular density in fibroids relative to endometrium [34]. Obviously, an extended microarray analysis between different location fibroids, its pseudocapsules, and adjacent endometrium need to be performed. Pseudocapsules at different locations need to be examined as different tissues. Given current data, pseudocapsule angiogenesis is increased, even more than nearby myometrium [35]. From these data this is mandated from myometrium but not the fibroid, while MED12 sequence results between pseudocapsule and fibroid, indicate the nontumor origin of the pseudocapsule [24]. In addition, solitary and multiple tumors should be analyzed in different sets, because multiple fibroids originate from MED-12 associated mechanisms while this is not the case for solitary ones [36].

**Figure 3:**
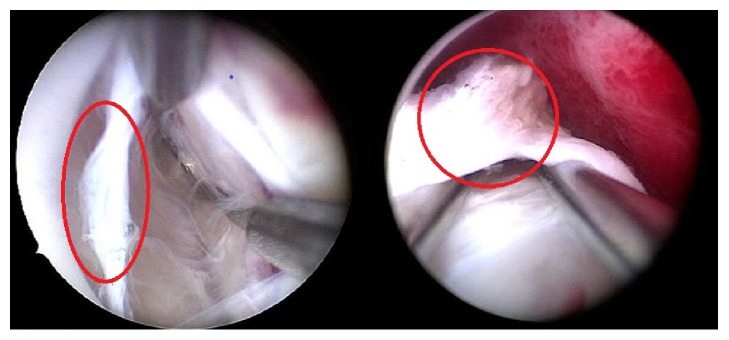
Myoma pseudocapsule in white evidenced by a red circle during a COLD LOOP hysteroscopic myomectomy.

For the importance of FP in myometrium muscle physiology, in case of submucous LMs, the surgical treatment could be not adequate in FP sparing, to save the LMs neurovascular bundle. Considering that the FP should be preserved during myomectomy procedure, the classical hysteroscopic slicing in the context of myometrium could not ensure a “myometrial sparing” approach and therefore the integrity of its pseudocapsule. Recently the “*cold loop*” hysteroscopic myomectomy was reported as a safe and effective procedure for the removal of submucous LMs with intramural development. Such technique allows identifying and sparing the FP (Figure 3) and the surrounding healthy myometrium mechanically, cutting the connective bridges of the FP anchoring the LM to the myometrium, without electricity use [6]. In a retrospective analysis of a large cohort of patients who underwent cold loop myomectomy, Mazzon et al. reported a postsurgical synechiae rate of 4.29%, of which 3.94 were light synechiae removed with the tip of hysteroscope during the follow-up hysteroscopy, 2 months after the surgery. The authors reported that preservation of FP and of myometrial integrity was associated with very few surgical complications and with enhanced healing, reducing risk of uterine rupture, and good fertility rates and delivery outcomes [37].

**Figure 4:**
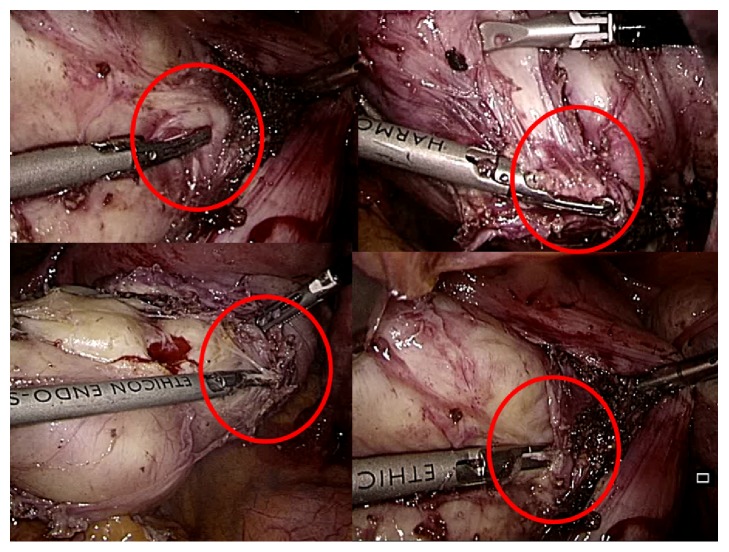
The pseudocapsule of the myoma is highlighted in the red circle: it is incised and cleaved from fibroid with a surgical instrument (to enucleate only the fibroid), preserving the myometrium below the pseudocapsule.

Concerning intramural LMs, studies have already been published that highlight the importance of intracapsular technique to preserve the myometrium integrity during enucleation of LMs, sparing pseudocapsule (Figure 4) [5, 31]. At the light of the study results and of previous report [38], the authors affirmed that the FP should be always preserved, as much as possible, during the myomectomy procedure, to have a better myometrial cicatrization and a better outcome on successive fertility [17, 31].

## 6. Conclusions 

Considering the increasing interest on the LMs and their fertility implications, the FP evaluation could open new perspectives in clinical research and the treatment of uterine myomas, due to its neuroendocrine and biological role on myometrium and on postsurgical myometrial healing. Our results confirm the relevant difference of FP thickness, particularly thickened under the endometrium for submucous LMs. As the submucous LMs are scientifically largely described as cause of sterility and infertility, FP should be more investigated for possible importance of its preservation in order enhance fertility, also in correlation to postmyomectomy healing and avoiding, i.e., intrauterine adhesion. Future studies should be focused on the correlation between the LMs volume and FP thickness, its amount of neurofibers, and its role on medical, surgery, and fertility outcomes.
